# A Qualitative Study of the Implementation and Continued Delivery of Complete and Partial Smoke-Free Policies Across England’s Prison Estate

**DOI:** 10.1093/ntr/ntac296

**Published:** 2023-01-11

**Authors:** Leah Jayes, Jessica Waddingham, John Britton, Rachael Murray

**Affiliations:** Institute of Health and Allied Professions, Nottingham Trent University, Clifton Campus, Nottingham NG11 8NS, UK; JJR Macleod Centre for Diabetes, Endocrinology and Metabolism, NHS Grampian, Aberdeen, UK; Population and Lifespan Sciences, School of Medicine, The University of Nottingham, Nottingham, UK; Population and Lifespan Sciences, School of Medicine, The University of Nottingham, Nottingham, UK

## Abstract

**Introduction:**

In the United Kingdom, smoking among prisoners is up to five times more prevalent than the national average. Between 2015 and 2018, HMPPS introduced a complete smoke-free policy in all closed prisons, and a partial policy permitting smoking only in smoking shelters in open prisons.

**Aims and Methods:**

This study aimed to explore the views of stakeholders regarding the implementation and continuation of smoke-free policies, including the management of nicotine addiction during imprisonment and after release. Individuals with key strategic and/or operational roles in delivering smoke-free prison policies across England were purposively sampled to complete a semi-structured interview. Twenty-eight interviews were analyzed thematically.

**Results:**

The smoke-free implementation across the closed prison estate was viewed as a success, though there were reports of reduced availability of smoking cessation support since the roll out. Participants thought the majority of tobacco smokers living in closed prison environments were now using an electronic cigarette, typically as a temporary means to manage nicotine addiction until release. In open prisons the partial policy has been less successful; high rates of smoking resumption on moving from closed to open conditions were reported, with many participants arguing that the open estate should also go completely smoke free. It was envisaged that most prisoners would resume smoking on community release.

**Conclusions:**

The smoke-free policies provide a unique opportunity to promote lifelong cessation in this highly disadvantaged group. However more could be done to adopt a consistent smoke-free policy across all prisons, and to support prisoners in quitting smoking and nicotine use during and after imprisonment.

**Implications:**

Our results identify the urgent need for more work to explore rates and reasons for relapse to smoking on transfer to the open estate and after release. With the majority of smokers in the closed prison estate now using e-cigarettes to manage their nicotine addiction, one way to support long-term tobacco abstinence could be to place greater emphasis on this switching behavior as a way of reducing tobacco-related harm within this population.

## Introduction

Worldwide, the prevalence of smoking among prisoner populations is between 2 and 8 times higher than in the general population,^1^ and smoke-free policies have been increasingly widely introduced to protect staff and prisoners from exposure to high levels of secondhand smoke (SHS). Jurisdictions rolling out smoke-free policies include Canada,^2^ most states of the United States,^3^ Australia,^4^ New Zealand,^5^ and more recently England and Wales ^6^ and Scotland.^7^ Akin to mental health settings, research suggests “complete” smoke-free prison policies (no smoking allowed within the perimeter wall, inside and outside of buildings by prisoners and staff) tend to be more successful than partial smoking restrictions (smoking allowed by prisoners and sometimes staff in 1 or 2 areas within the prison perimeter, usually prison cells or outside exercise yards).^8,9^

Before smoke-free policies were introduced in the United Kingdom, smoking prevalence amongst prisoner populations was estimated at between 70% and 80%,^10–13^ five times the national average ^14^ The average age of death for prisoners dying of natural causes in England and Wales is 56 years, 25 years less than in the general population.^15^ Smoking is a significant contributor to this low life expectancy.

Her Majesty’s Prison and Probation Service (HMPPS) in England and Wales cover 118 prisons currently holding 79 000 male and female prisoners.^16^ (See Supplementary File 1 for information on prison types and categorization within HMPPS). Over a 3-year period starting in 2015, smoke-free policies were introduced across HMPPS,^6^ with a complete smoke-free policy imposed on the 103 closed prisons (Categories A, B, and C) and a partial policy, permitting tobacco smoking on the prison sites within designated shelters, in the 15 open prisons (Category D). Prisoners who smoked were offered smoking cessation support including up to 8 weeks of behavioral and pharmacological support with Nicotine Replacement Therapy (NRT), such as patches or lozenges.^17^ Prisoners were also able to purchase electronic-cigarettes (e-cigarettes) from the prison shop (known as “canteen”), one being a single-use disposable product and the other a rechargeable vape pen with pre-filled capsules containing up to 18 mg of nicotine and available in several flavors. Within the closed prison estate prisoners are only permitted to vape in their cell whereas in the open estate, alongside being allowed to vape in their cell, prisoners are also permitted to vape outside in designated vaping shelters.^18^

Complete smoke-free policies have generated significant reductions in levels of SHS in prisons^19–22^ but their potential as a means to reduce long-term smoking among current and former prisoners has been little investigated. This study aims to explore stakeholders’ views on the implementation and delivery of complete and partial smoke-free policies, specifically in relation to the management of nicotine addiction throughout a sentence and after release.

## Methods

### Design

A qualitative study was conducted using semi-structured one-to-one interviews, underpinned by a constructionist research paradigm.^23^ This study follows the Standards for Reporting Qualitative Research.^24^

### Participants and Recruitment

Sampling was purposive and guided by principles of information power.^25^ Those tasked with implementing smoke-free policies nationally working within HMPPS Headquarters and regionally within Public Health England (PHE) were identified by the lead researcher (LJ), contacted, and invited to participate. At an establishment (local) level, interviews were completed with HMPPS and Healthcare staff working in 3 male prisons; selected to provide variety in relation to smoke-free policy, security level, size, prisoner occupancy, and the typical length of stay (for study prison characteristics see Supplementary File 2). Individuals who had a lead role in introducing and/or continued delivery of smoke-free policies were identified to researchers by the prison Governor/Deputy Governor and Head of Healthcare. All staff identified were approached by LJ to discuss the project and all consented to take part. Preliminary analysis (LJ and JW) concluded that our sample size of 28 held appropriate information power for recruitment to cease. Interviews were completed between August and November 2019, roughly 4 years after open estate introduced the partial smoke-free policy and 16 months after the last closed prison implemented a complete smoke-free policy.

### Data Collection

We used one-to-one interviews, either face to face at one of the three establishments sampled or over the telephone to capture the variation of experiences linked to individual job roles within different organizations. The semi-structured interview guide was developed through reflections on LJ’s work and continued involvement in the introduction of smoke-free polices in English prisons^26^ in conjunction with a review of existing literature. The guide covered the participant’s role and involvement in prison tobacco policies, reflections on the implementation of smoke-free policies and ongoing delivery, to include the management of nicotine addiction in prison and after release (Supplementary File 3). The interviewer adjusted the order and context of topics covered in the interview guide as appropriate (eg, national or establishment level), prompted further discussion where relevant and invited the interviewee to raise any points they felt were pertinent to the topic. Participant information on age, gender, smoking status, and years working within the current organization was also collected. Interviews typically lasted for around 45 minutes. No incentives were offered for participation. With written consent from participants, interviews were digitally audio recorded and then transcribed verbatim by a professional transcription company.

### Data Analysis

Thematic analysis was used to analyze the data;^27^ selected for its flexible approach to both allow themes to be identified inductively from the data but also those pertained to the aims of the study.^28^ Transcripts were checked and de-identified by two researchers (LJ and JW), aiding familiarization. LJ and JW then independently coded a selection of transcripts before meeting to discuss initial ideas around potential themes. Next, the researchers independently coded a further set of transcripts before meeting again to construct and map their “working” themes (and subthemes). Due to the lead authors’ familiarity with the area (and possible preconceptions) and as part of our reflexive practice, a third researcher (RM) checked 30% of transcripts for consistency and interpretation against the developed thematic map. NVivo 11 (QSR International 2016) was used to manage and organize the data.

## Results

### Participant Characteristics

Twenty-eight participants involved in delivering smoke-free policies at strategic and/or operational level were interviewed (Table 1). Eighteen were HMPPS employees who either worked nationally at HMPPS Headquarters (*n* = 5) or locally (*n* = 13) within one of the three study establishments. At the time of interview, all except one individual working in HMPPS Headquarters were still in dedicated roles related to the continued delivery of smoke-free policies throughout the English prison estate. Of the 13 HMPPS employees working locally, 4 worked within the open prison and 9 in the 2 closed prisons sampled, and all those working in the closed prisons had played a role in delivering the smoke-free policy within their prison. The open prison did not have a designated team set up for implementing their partial policy roll out. Roles of the HMPPS staff interviewed at the three prison sites included; Governor, Deputy Governor, members of the senior management team, custodial managers, and prison officers.

**Table 1.** Participant Characteristics (*n* = 28)

Characteristic	Number
Mean age (range)	48 years (24–59 years)
Sex	
Male	9
Female	19
Smoking status	
Never smoker	17
Ex-smoker	7
Current smoker	1
Current e-cigarette user	3
Mean number of years working within current organization (range)	19 years (1–37 years)
Employing organization	
Public Health England	2
Healthcare provider (NHS Trust or private)	8
HMPPS Headquarters	5
HMPPS Establishments (prisons 1–3)	13

The 2 PHE employees interviewed were regional Public Health Specialists for Health and Justice, one of which worked on the national delivery of smoke-free prisons and the other supported the move across the 3 study sites. Healthcare staff interviewed (*n* = 8) were employed by 1 local NHS Trust (covering 2 prisons) or 1 private Healthcare provider (covering 1 prison) with interviews being conducted with Heads of Healthcare and Mental Health Teams, senior nurses, pharmacy technicians, and healthcare assistants. All healthcare staff interviewed managed or delivered prison smoking cessation services pre- and/or post-policy implementation.

With the exception of 2 staff members, all HMPPS and Healthcare staff working within the 3 establishments (Prison 1 = 6; Prison 2 = 7; Prison 3 = 8) had been working in their respective prisons prior to the smoke-free policies being introduced.

Five inter-related themes are presented below. One theme was drawn from data collected from staff working directly in or across the closed prison estate only (Closed estate: Reflecting on the implementation of a complete smoke-free policy; *n* = 20) and another from those working in or across the open estate only (Open estate: delivery of and debate over a partial policy; *n* = 15). The remaining three themes were obtained from information across the whole dataset. Illustrative quotes are provided in Boxes 1–5 alongside participant characteristics: participant ID, employing organization (national and establishment level HMPPS staff [HMPPS] or those working for a healthcare provider within an establishment or regional PHE lead [Health]), smoking status (never smoker [NS], ex-smoker [ES], current smoker [CS], e-cigarette user (EC]), and prison type for those working directly in an establishment ([Closed] or [Open]). A thematic map demonstrating the relationships between the themes is provided in Supplementary File 4.

### Closed Estate: Reflecting on the Implementation of a Complete Smoke-Free Policy

When reflecting upon the reason behind the closed estate moving smoke-free, most participants outlined that the policy had been introduced to reduce SHS exposure within prison sites. From the perspective of those within HMPPS Headquarters and Senior Management across the three prison sites this was driven by the need to reduce potential litigation from prisoners and staff members, whereas others interviewed assumed the move was related to potential health benefits for all.

#### Success of the Implementation

Everyone referred to apprehension that disorder, drug use, and self-harm would increase as a result of the smoke-free policy, and many were surprised by how smoothly the transition went, with very low levels of disorder or disruption. In light of this, and alongside a clear reduction in SHS (as observed first hand by many interviewed) the majority of participants concluded that the move to smoke-free across the male and female closed estate in England had been a big success (Box 1; 1a).

**Box 1. Closed estate: reflecting on the implementation of a complete smoke-free policy**

1a.* To be trying to take an unstable prison population smoke-free during that time was potentially, could potentially be catastrophic and I think we managed it exceptionally well because we didn’t have really any significant incidents that could be totally and directly linked to smoke-free….so I think we did remarkably well. *P03-HMPPS-Nat-NS

1b. * I don’t know, it’s hard for timings but I know, I think once that came in to effect [smoke-free policy], I think there was probably maybe an increase in demand for spice and other drugs, so I’m not sure that we can say that, “Oh because of that [smoke-free policy], we then caused ourselves a drug problem”, it’s always going to be supply and demand and it’s going to be another illicit item, isn’t it? But, I’d say there was probably a peak, I think most establishments would say that there was an increase and then it slowly levelled out a little bit. *P21-HMPPS-Closed-NS

1c*. You walk past their cells at night and there are people in those cells smoking. What they’re smoking, anyone’s guess. But sometimes you go, “Oh, they’ve got a proper fag”, and, “Oh god, I don’t like the smell of that one”…..tobacco is highly sought after and it does get a lot of money. *P24. Health-Closed-CS

People attributed this success to several aspects of the roll out; a long lead time; sustained communication to staff and prisoners; pre- and post-policy intelligence gathering (eg, on disorder, self-harm, trading); increased provision of smoking cessation services (delivered by healthcare providers); the establishment of dedicated smoke-free delivery teams within each prison to implement the policy; a collaborative approach and shared learning across the prison estate and between healthcare providers, PHE and HMPPS; and the hard work and dedication of all those involved. Although all of these aspects played a part in the success of the policy, it was the introduction of vaping devices that was commonly cited as a “game-changer” or “savior” in the policy introduction (see theme “The introduction and popularity of e-cigarettes”).

#### Impact of the Policy

Nearly everyone mentioned how prisons now felt cleaner and healthier environments to work and for prisoners to live in. Many felt that there had been a reduction in fires since cigarette lighters had become a prohibited item during the roll-out. There was a perception by some that levels of self-harm and drug use increased immediately after the policy implementation but that these levels soon returned to normal (1b). Participants spoke about how the move to smoke-free had led to tobacco becoming an illicit (“black-market”) item alongside other drugs, and this led to an increase in its value. Participants said it was clear that on occasions tobacco was being smuggled into prisons as staff could smell it. Smoking make-shift cigarettes generated from NRT products, tea bags, banana skins, or bible paper was also reported (1c).

### Open Estate: Delivery of and Debate Over the Partial Smoke-Free Policy

#### The Partial Policy in Practice

As in the closed estate, participants thought the partial policy was introduced to reduce SHS exposure to prisoners and staff. However, in contrast to the closed estate, those working within the open estate described little build up or publicity in advance of going partially smoke free. They explained how the partial policy, which allowed smoking and vaping only in outdoor shelters, did not work as smoking and vaping still occurred throughout the prisons and this was extremely hard to police. Most notably, participants reported that smoking and vaping would occur on the wings or in cells overnight when prisoners were prevented from accessing the shelters.

#### Returning to Smoking on Transfer to the Open Estate

Participants estimated that around 70–90% of prisoners who had smoked prior to entering prison began smoking again when they moved from the closed to the open estate. Those working in the open prison outlined how reception staff would ascertain smoking status from prisoners on arrival from a closed prison and, as was the practice before going smoke free, offered them a “smokers pack” consisting of loose tobacco, rolling paper, and a lighter. Some participants described this practice as frustrating and felt it encouraged the immediate resumption of tobacco smoking (Box 2; 2a).

**Box 2. Open estate: Delivery of and debate over the partial smoke-free policy**

2a.* Yeah, as a healthcare professional, it’s a bit, you get a bit exasperated because they’re coming in from a closed prison and a lot of people don’t want to vape, so they give up and then they’re coming here and we know quite freely that the prison are offering them a smoking pack which is free … .They’ll come in to reception and I’ll be shouting them in to reception and then they’re outside having a fag because they’ve got their [smokers] pack and they’ve done a roll-up and they’re outside smoking. *P17-Health-Open-NS

2b.* So when they come here from a closed establishment, they’ve come to an open establishment, and they might have been in there [in a closed prison] for 10 years where they’ve had the door open and closed for them, told to get up and not, have their lights switched on and off, and they’re here [an open prison] and they’re like, “oh my god!”. So you can understand why they might reach for the tobacco straightaway, because that’s the only comfort that they’ve then got that might make them feel a bit more better. *P08-Health-Open-NS

2c.* If I’m giving my personal opinion here, I think we missed a trick there. I think we should have made the open estate smoke-free as well….because we have people who have gone through a smoke-free project in the closed estate, we have hopefully made a significant number of them smoke-free and then we put them back in to a smoking environment whilst they’re still in our care and that just doesn’t feel right to me. I think we could have made it smoke-free, yes we could not have stopped them from purchasing cigarettes when they went out on release on temporary licence, but we could have stopped them bringing those in to prisons and smoking it whilst in prison. *P03-HMPPS-Nat-NS

2d.* We can’t realistically have a smoking ban in an open prison because you can’t police it when somebody’s outside and they’re out there on licence, they’re doing whatever they’re doing for the day, the week, the month, whatever it is and if they wish to smoke that’s a permitted activity outside; we can’t then expect them to come in and then not smoke when they’re inside prisons. *P04-HMPPS-Nat-NS

Those working in the open prison reported that many prisoners found the transition between closed and open conditions stressful, as they were moving from a regimented regime into one which was more relaxed with increased personal choice, and therefore felt it was inevitable that prisoners would accept the offer of tobacco on arrival as a way to cope (2b). Participants also thought it must be hard for prisoners to maintain their tobacco-free status in the open prison environment as the majority of people living around them smoked. It was estimated that around 30% of prisoners in the open prison were e-cigarette users, but that most of these also smoked tobacco.

#### Overall Support for a Complete Smoke-Free Policy in the Open Estate

A majority of participants expressed support for making the open estate in England completely smoke-free. On reflection, participants felt that not extending the complete smoke-free policy into the open estate during the national roll out was a missed opportunity (2c). There were however some participants working in this setting who thought the policy in the open estate should remain, believing it to be impossible to prevent tobacco from coming back into the prison after prisoners returned from working in the community or day/overnight release, and unfair on those prisoners who choose to smoke while out in the community (2d). They felt that open prisons, where possible, should replicate the community, and therefore smoking should be permitted. Those in support of a complete smoke-free policy throughout the open estate thought these views were unconvincing and dismissed claims that a smoke-free policy would be hard to manage, and suggested that tobacco could be treated like any other prohibited item (eg, mobile phones) and handed over on return to the prison from authorised leave.

### Continued Delivery of Smoking Cessation Services Throughout the Prison Estate

#### Current Smoking Cessation Provision

Those working at regional and national levels said healthcare providers throughout the whole estate should be offering smoking cessation services to prisoners throughout their sentence in line with what is set out in the Minimum Service Offer for smoking cessation.^17^ They went on to add that they had become aware of providers who had failed to meet these standards since the policy introduction (Box 3; 3a).

**Box 3. Continued delivery of smoking cessation services throughout the prison estate**

3a. *I’m aware of what they should do [in terms of smoking cessation offered] and the minimum service offer and how that should be worked through and ensuring that’s maintained. I’m also aware that poor old [colleague name] is forever trying to blow that one back up again because we’re starting to get maybe things perhaps not done as well as they should do. *P04-HMPPS-Nat-NS.

3b. *They’re [prison staff] just automatically giving everybody who comes through the door, a vape……So, by the time they get to see healthcare, they’ve already gone through all the prison process, and then they get into healthcare to see the nurse for their reception screen, where they would be given patches in reception if they needed them … .But by the time they get to see the nurse and say to them, “Are you a smoker? Yeah, but I’ve got a vape, miss” and that’s the end of it. *P24-Health-Closed-CS.

3c. *We haven’t got smoking cessation here. It’s deemed that when a man gets convicted he’ll go to a local/reception prison, they will support him through smoking cessation because they should be smoke-free. So by the time he comes here [training prison] he either has had smoking cessation, or a vape, or both. *P01-HMPPS-Closed-ES.

3d. *We’ve only got one person on the smoking course at the moment. But this time last year when I first started we had like 15 people but it’ll go in swings and roundabouts. So when people are coming towards the end of their sentence and they’ve done a long time in they’ll go, ‘oh I want to quit now’ and they’ll come to me.….. most of them don’t want to be smoking when they go out, and once they get a job on the outside they’re like *‘*oh, I don’t want to be spending my money on that, I want to be saving for this, that and the other’. Or they’ll be doing a driving job where they can’t smoke so they’ll just come to me. And they’re probably the ones that find it the easiest to quit, to be fair. *P08-Health-Open-NS

#### Closed Prisons

The prison staff working in the “local” prison (Prison 1) were under the impression that prisoners could only enroll on the smoking cessation course when they first arrived at the prison, though healthcare staff explained that prisoners could access the courses throughout their stay but that this was not advertised to prisoners. Healthcare staff working within the local prison outlined how there was little to no uptake of smoking cessation support by prisoners on arrival because most of them had already accepted an e-cigarette offered by prison staff earlier in the reception process (3b). In addition, healthcare staff reported that prisoners were only eligible to complete the smoking cessation course if they were not using an e-cigarette, due to concerns over the dual use of e-cigarettes and NRT and for fear of prisoners trading NRT products issued while continuing to use an e-cigarette. Staff within the “training” prison (Prison 2) explained how prisoners residing there should have already accessed any cessation support they required earlier in their sentence and therefore their Healthcare provider did not offer any cessation support (3c).

#### Open Prison

Those working in the “Category D” site (Prison 3) said their smoking cessation course had remained the same throughout the partial policy roll out; they offered a 12-week course which included one-to-one behavioral and pharmacological (NRT or Varenicline) support. Healthcare staff commented that uptake of the course fluctuated, with those nearing release and working in the community most regularly signing up and having the most success (3d).

#### Role of E-cigarettes Within Smoking Cessation

Two participants working in Headquarters outlined how prior to the introduction of smoke-free policies they had been told e-cigarettes could not be incorporated into the healthcare-run smoking cessation course as the advice they followed (National Centre for Stop Smoking Training ) did not support the use of e-cigarettes as an alternative to NRT. Many staff felt the exclusion of e-cigarettes from prison smoking cessation required revisiting (see theme “Impact of the smoke-free policies on long-term smoking abstinence”).

### The Introduction and Popularity of E-cigarettes

The introduction of e-cigarettes was viewed by many as crucial in the successful roll-out of the smoke-free policy in the closed estate, helping prisons to maintain order (Box 4; 4a).

**Box 4. The introduction and popularity of e-cigarettes**

4a. *I don’t say I dislike them [e-cigarettes] because they help me keep this place settled. So from a prison service point-of-view they [e-cigarettes] were quite a good instrument and a good tool to allow us to keep control of establishments in the middle of summer when we introduced the smoke-free policy … Because we gave up smoking in the middle of summer, and summers are tricky in establishments at the best of times. *P01-HMPPS-Closed-NS.

4b. *I’d say a lot of them [use an e-cigarette] … I suppose it would be the same as how many smoked because they literally have swapped one for the other because it’s exactly the same. They are getting that hit, it’s hand to mouth and they can put stuff [illegal substances] in it….so we had quite an increase in secondary exposure on PS [Psychoactive Substances] around summertime because they were adapting the [vape] pens. ****Interviewer: Prisoners were somehow putting PS into the vaping device? ****Yeah and, of course, if you’re smoking PS it’s wrapped up with tobacco, it’s easier to spot, but with a vape it was vapour and it was drifting around the wing and staff were affected quite badly.* P18-Health-Closed-ES.

4c. *Tobacco used to be a form of currency, a large form of currency. A lot of debt associated with it, a lot of self-harm directly associated with tobacco, or the lack of it, and vapes became the new tobacco. It was a form of currency. We put in within our document policy that they [prisoners] could only have so many [vape] cartridges, so they [prisoners] shouldn’t have no more than 10 cartridges. *P14. HMPPS-Closed-NS.

4d.* But, my views, on the vapes I don’t think, there’s not been much scientific evidence and we don’t really know whether you’re actually doing more harm vaping than by tobacco, there’s not, for me there’s not enough stringent evidence that one outweighs the other so for me. *P17- Health-Open-NS

**Box 5. Impact of the smoke-free policies on long-term tobacco abstinence**

5a. *Was it successful because we didn’t lose many prisons, or people didn’t get hurt. Yes. If you’re saying is it successful for reducing cancer and people stopping smoking? Not really. It’s just a stop-gap, because they’re still smoking and all that … It’s just a stop-gap, it’s just like having six months off it. *P01-HMPPS–Closed–ES.

5b. *I suppose the other bit for me is that every prisoner I’ve spoken to has an intention to start smoking on release. Whether they do or not I don’t know, but they’re very clear that that’s what they’re going to do, even though you might have a conversation with them about why would you do that. “You’ve been a year without a cigarette, why would that be the first thing you do?”* P11-HMPPS-Nat-ES.

5c. *No, I just, you just keep remembering all we’ve done is forced people to stop smoking in prisons and that’s as far as our remit went … Whereas when I was ready [to stop smoking], I did that and I did that through choice, an informed choice, but I did that myself, but we don’t give our lads that choice. We don’t give them any of that, we just say, “No, you’re not smoking.” And it doesn’t work because, clearly, they come to Cat D and smoke again.* P04-HMPPS-Nat-NS.

5d. *It’s a myriad of things. But I think it’s social/peer pressure as well. “I’ve stopped smoking”, “Don’t be silly, have a fag, you’ve just been released”. Or they want to smoke. Mum and dad smokes, brother and sister, everybody smokes, so they have a fag and that’s what they’re brought back into. Or coping strategies. The majority will go out in the end and have a pint and a drink and a cigarette. So it’s more the social part and the relief of being released. The celebration or the, “Christ, I’ve been realised, I can’t cope, therefore I need to smoke”. So the pressure of being released or the joy or being released.* P01-HMPPS-Closed-ES

#### Estimates of Use and the Practice of Nicotine Maintenance

As previously described, on arrival to prison most smokers accepted an offer of an e-cigarette in preference to a smoking cessation course. Within the closed estate it was estimated that around 70–80% of prisoners were now regular users of rechargeable e-cigarettes. Some participants thought that tobacco smokers coming into prison were simply switching over to e-cigarettes as a way to manage their nicotine addiction until release (4b).

#### E-cigarettes as the “New Tobacco”

Many people said the issues historically relating to tobacco smoking prior to the complete smoke-free policy in the closed estate had simply shifted onto e-cigarettes, resulting in the pre-filled e-cigarettes capsules becoming a new form of currency and leading to debt, bullying, and a vehicle for drug use (4b); a couple of staff working in the closed estate gave examples of instances of prisoners vaping illegal substances. As e-cigarette capsules soon became currency once the smoke-free policy was introduced, prisons had to introduce a limit on the amount of e-cigarette capsules a prisoner could purchase each week from the canteen (4c). Staff working in the closed prisons outlined how the policy stated that e-cigarettes could only be used in prisoners’ cells, but that they were often used in other areas of the prison.

#### Concerns about the use of e-cigarettes

Some participants were concerned that some e-cigarette users were prior nonsmokers who used e-cigarettes to look “cool” or to “fit in”. Some staff were also concerned that most prisoners used 18 mg nicotine capsules in their e-cigarettes, regardless of how much they had previously smoked. A couple of staff thought this was because prisoners simply continued to use 18 mg strength capsules having initially only been offered this strength on arrival to prison and a couple believed that 18mg capsules were most regularly traded amongst prisoners (with lower strength capsules being perceived as having a lower monetary trading value) and that was why they were most commonly purchased and used. Some questioned the safety of e-cigarettes (4d).

### Impact of the Smoke-Free Policies on Long-Term Tobacco Abstinence

#### Estimates of Smoking Relapse After Prison Release

Nearly all participants thought the complete and partial policies had little impact on likely future smoking by prisoners and hence long-term prisoner health (5a). However, some participants pointed out that long-term smoking abstinence was never the aim of the policies.

Participants estimated that most prisoners who had smoked prior to entering prison would resume smoking quickly on return to the community or transfer from a closed to an open prison. Staff said on occasion they would speak to prisoners who were grateful for the opportunity to stop smoking in prison, however more often, they said conversations with prisoners would revolve around their plans to return to tobacco either once transferred to open conditions or on release into the community (5b). Several participants expected that those on longer sentence to have the best chance of lifelong abstinence (compared to shorter sentences) since they will have had longer in a closed smoke-free environment and had more opportunities to engage with cessation services.

#### Barriers to Maintaining Tobacco Abstinence After Release

Participants thought the nature of enforced abstinence in the closed estate was not conducive to long-term cessation. It was felt that because prisoners had been forced rather than chosen to stop smoking they would return to smoking once permitted (5c); indeed, two participants believed exercising this choice through resumption of tobacco smoking was a way for prisoners to “stick two fingers up” at HMPPS. As previously mentioned, many believed prisoners were using e-cigarettes as a way to maintain their nicotine addiction whilst in prison and that few of these would continue using an e-cigarette after release. Staff reported that few prisoners took their prison e-cigarette home, but recognized that giving or selling possessions was often a rite of passage before release.

Some staff reported that the return of tobacco seized on arrest at the time of release, or prisoners being collected at the prison gate by friends or family who bring along tobacco, facilitated the return to smoking. Some thought prisoners simply enjoyed smoking tobacco and therefore would resume as soon as possible. As with the transfer from the closed to the open estate, several participants said that for many prisoners release into the community was very stressful, and this would result in many turning to tobacco as a way to cope or in some cases to celebrate the release (5d). Participants also expected relapse to smoking to be associated with the resumption of alcohol or other drug use.

Many participants felt the biggest influence on tobacco smoking after release would be the people and environments people returned to. They expected the prevalence of tobacco smoking to be high amongst the families and friends of prisoners and that these groups might exert pressure on them to smoke tobacco again (5d). Added to this, participants outlined how a proportion of those released would enter hostels or become homeless, where again, they envisaged the majority would smoke, making it extremely hard to remain tobacco free after release.

## Discussion

### Main Findings

This qualitative study found that the introduction of a complete smoke-free policy within the closed prison estate in England was considered to be a success in terms of safe implementation, and reducing smoking and exposure to SHS. The availability of e-cigarettes was identified as a key contributor to this success, though problems previously associated with tobacco such as trading, bullying, debt, and use as a vehicle for drugs had now shifted onto e-cigarettes. There appeared to be limited access to and uptake of the healthcare-run smoking cessation course. Of those working directly in or across the open estate, the majority supported moving from a partial to a complete smoke-free policy, citing the high rates of smoking resumption on arrival in open conditions and the impracticalities of policing the current partial policy. Participants thought that most prisoners would return to tobacco smoking once they were legally allowed, in an open prison or in the community, to do so. Participants outlined several reasons, experienced in prison and/or after release, for resumption of tobacco smoking after release, including personal (stress-relief), inter-personal (prison issue e-cigarettes left behind, family/friends providing tobacco on release), structural (lack of prison cessation services, property containing tobacco returned at release), and environmental factors (returning to communities/housing where the majority of people smoke).

### Discussion of Findings

Research from other countries suggests that complete smoke-free policies, whereby no smoking is allowed within the perimeter wall, tend to be more successful in terms of lowering levels of SHS and in management and enforcement, than partial smoke-free restrictions allowing smoking by prisoners and sometimes staff in cells or designated outdoor areas.^8,29–31^ Our findings support this, with participants working in the closed estate commenting on observed reductions in SHS and a few instances of tobacco contraband whereas those working in the open estate found the partial policy to be ineffective in moving smoking and vaping to designated outdoor shelters. This, alongside concerns about the high rate of resumption to tobacco smoking on transfer from closed conditions, led to the majority of those working in or across open prisons to outline their support for a complete smoke-free policy throughout the open estate in England. Further work is required to explore rates of relapse and reasons behind it as to the authors’ knowledge there are no other global jurisdiction where prisoners can move between complete and partial smoke-free policies whilst on the same sentence.

Prisons in England, Wales, and Scotland are unusual in allowing prisoners to purchase e-cigarettes as a way of managing their nicotine addiction in smoke-free prisons, and these have proved highly popular. Concerns relating to the use of e-cigarettes as a new form of currency and a vehicle for drug use were raised in Scottish smoke-free prisons.^32,33^ Our findings suggest that e-cigarette capsules have replaced tobacco as a form of currency in English prisons. As other jurisdictions with complete smoke-free policies have found, removing tobacco from a penal system does not eliminate its use as a currency and the associated issues altogether, instead a monetary value is placed upon something else deemed useful or desirable to prisoners; for example, in one U.S. state the prison currency shifted from tobacco to ramen noodles due to the poor quality and quantity of prison food.^34^

Those working nationally acknowledged that since the smoke-free policies had been introduced they had become aware of occasions where prison healthcare providers had not met the Minimum Service Offer (MSO) for smoking cessation.^17^ To some extent this appeared to be the case within the two closed prisons sampled, with smoking cessation only being available to those entering prison. Within this study the two main options prisoners had to support their nicotine dependence, e-cigarettes or accessing smoking cessation services, were spoken about as distinct options; HMPPS distributing e-cigarettes via the canteen provider and smoking cessation being the responsibility of the healthcare providers. However several of our participants believed e-cigarettes should be incorporated within the health providers smoking cessation service. Since nicotine-containing e-cigarettes have been shown to be more effective than NRT for successfully quitting^35^ and are recommended for treating tobacco dependence within National Institute for Health and Care Excellence (NICE) guidelines,^36^ this seems an appropriate proposal.

Findings from the U.S. and Australia suggest that the benefits of complete smoke-free prison policies are largely confined to prison settings, with 60% of former (pre-prison) smokers relapsing within a day of prison release and almost all within 6 months.^37–40,8^ This was largely the view of participants in this study, who expected little long-term impact on abstinence after release or transfer to open conditions. With the majority of smokers on entry to prison moving to an e-cigarette during their sentence, one might assume some prisoners might continue to use an e-cigarette after release. However, staff in this study felt e-cigarettes were mainly used as a way for prisoners to maintain their tobacco addiction until they were permitted to smoke tobacco again. Since UK research suggests that over half of prisoners want to stop smoking,^41,42^ providing education on the use of e-cigarettes as a cessation device (and not just a maintenance tool) could help to prevent smoking relapse in this population.

Potential predictors of post-release smoking outlined in this study correspond with findings from research completed in the U.S. and Australia where participants have been followed up after their release. Identified reasons for relapse include a demonstration of resistance to enforced abstinence while in prison;^43^ belief that tobacco would alleviate the stress associated with release;^43,44^ finding smoking pleasurable;^45^ resumption of substance misuse;^46^ smoking being associated with feelings of/or celebrating freedom;^43,47^ and returning to homes, housing facilities, or social environments where smoking is prevalent.^43,44^ Additional reasons for smoking relapse which appear to be specific to the prison system in England and outlined in this study include the use of e-cigarettes as a nicotine maintenance tool, e-cigarette users not taking their prison issue device home, and tobacco confiscated on arrest being returned to prisoners at the point of release. No research has specifically explored the resumption of tobacco smoking on arrival to open conditions in England, however participants in this study anticipated similar reasons resulting in relapse on release to the community; easy access to tobacco (offered via “smoker packs”) on arrival, using tobacco as a stress relief due to changing circumstances, and entering an environment with high smoking prevalence.

### Strengths and Limitations

A strength of this study is that our participant group worked in both HMPPS and the healthcare agencies that led the smoke-free implementation nationally and locally and continue to contribute to the ongoing day-to-day delivery of the policies. Alongside our qualitative approach, this study offers a rich and comprehensive account of smoke-free policies across a large national jurisdiction. Although the participant group has been highlighted as a clear strength of this study, the views of this sample (having led and influenced policy roll out) could have been biased, for example, in relation to the success of the policy implementation. Although interviewees working across the settings did outline some less favorable impacts of the policy introduction. It is also important to note that caution should be applied to percentage estimates provided by participants as there could be a degree of error in their estimation. This study sample does have a slight over representation of Females, given two thirds of the sample were Female and within HMPPS Females account for half of all staff employed.^48^ Differences in the way smoke-free prison policies in England are implemented compared to other criminal justice systems may in part limit the transferability of these findings to other global criminal justice systems. We are unaware of any other jurisdiction that has both complete and partial smoke-free policies within the same prison system and also very few prison systems currently allow the use of e-cigarettes. This said, this study does offer learning for other systems worldwide looking to move completely or partially smoke free, or introduce e-cigarettes.

### Implications for Policy and Practice

This study provides insight as to why prisoners may return to tobacco smoking once permitted and in turn highlights areas for potential focus in promoting longer-term cessation. Findings suggest nearly all smokers entering the closed smoke-free estate simply switched to vaping, with very little uptake in smoking cessation services (offering behavioral support and/or NRT). This could be attributed to a lack of cessation provision offered throughout a prisoner’s sentence or the fact that e-cigarettes were not offered as a cessation aid within the stop-smoking service; to the authors' knowledge little has changed since this study was completed. As a result, any future intervention to support post-release tobacco abstinence could place greater emphasis on maintaining this switching behavior as a way of reducing harm within this population. In doing so, practices around prisoners leaving behind their prison issue e-cigarettes at release would need to be explored alongside looking at the type of e-cigarette offered in prison and whether the complementary pre-filled e-liquid capsules could easily be accessible in the community.

## Conclusion

This study provides a comprehensive account of smoke-free implementation across HMPPS from the perspective of key stakeholders and highlights important areas where work is required to support those moving through the prison system in stopping smoking and nicotine use whilst in prison and after release. This work also reinforces what researchers in other smoke-free criminal justice settings globally have concluded, that there is an urgent need for high-quality research around strategies to reduce smoking relapse after release from smoke-free prisons.^45,49^

## Supplementary Material

A Contributorship Form detailing each author’s specific involvement with this content, as well as any supplementary data, are available online at https://academic.oup.com/ntr.

ntac296_suppl_Supplementary_Files

## Data Availability

The data underlying this article cannot be shared publicly to protect the privacy of participants. The data will be shared on reasonable request to the corresponding author.

## Patient and Public Involvement

This study is part of a larger programme of work, which in totality has been reviewed by two Patient and Public Involvement groups: a Criminal Justice PPI group (University of Plymouth) and the Tobacco and Nicotine Discussion Group (University of Nottingham).
