# Supplementary material for: A Qualitative Study of the Implementation and Continued Delivery of Complete and Partial Smoke-Free Policies Across England’s Prison Estate
Source: Nicotine Tob Res. 2023 Jan 11;25(6):1099–108. doi: 10.1093/ntr/ntac296 (PMC10202636; doi:10.1093/ntr/ntac296)
Supplement: ntac296_suppl_Supplementary_Files [file ntac296_suppl_supplementary_files.docx]

**Supplementary File 1**

**Categorisation of prisoners and types of prisons in England and Wales**

Adult prisoners (those aged 21 or over) are given a security categorisation (from A – D) according to the risk they present to security, safety and public protection (20). Most men entering prison will initially be held within a ‘local’ prison (holding both those on remand and sentenced) before, occupancy permitting, being moved to a prison providing an appropriate level of security (often a ‘training’ prison) (see Table 1). Due to the small female prisoner population, female establishments are categorised into either ‘closed’ or ‘open’, with all female closed prisons holding category A, B, and C prisoners. People in prisons can be released either from closed or open establishments.

Overview of prisons and corresponding prisoner security categories

| **Prison Type** | **Category** | **Prisoner characteristics** |
| --- | --- | --- |
| Closed prisons  (secured perimeter and a range of internal security measures)  (103 prisons) | A | Prisoners whose escape would be highly dangerous to the public or the police or the security of the state, and for whom the aim must be to make escape impossible. |
|  | B | Prisoners whose assessed risks require that they are held in the closed estate and who need security measures additional to those in a standard closed prison. |
|  | C | Prisoners who are assessed as requiring standard closed conditions, and do not need additional security |
| Open prisons (minimal perimeter and physical security features)  (15 prisons) | D | Prisoners who are either assessed as presenting a low risk or whose previously identified risk factors are now assessed as manageable in low security conditions. Tend to have longer sentences (+5 years) and in the male estate have worked down the prison security categories. |

Reference: Ministry of Justice, HMPPS. Security Categorisation Policy Framework. Available online: <https://assets.publishing.service.gov.uk/government/uploads/system/uploads/attachment_data/file/1011502/security-categorisation-pf.pdf> (accessed 25/11/21); 2021

**Supplementary File 2**

**Study prison characteristics**

|  | **Prison 1** | **Prison 2** | **Prison 3** |
| --- | --- | --- | --- |
| **Type, security category and function** | Male  Category B  Closed (local) | Male  Category C  Closed (training) | Male  Category D  Open |
| **Prisoner status** | Remand and sentenced | Sentenced only | Sentenced only |
| **Prisoner roll count** | 650 | 1100 | 581 |
| **Type of smoke-free policy** | Complete | Complete | Partial |

**Supplementary File 3**

**Interview guide**

Stakeholder Interview Topic Guide

Introduction

- Welcome and thanks; introduce yourself
- Give a brief background on the purpose of the interview
- Explain what will happen in the interview
- Reaffirm the interviewees right to withdraw and ensure that they are comfortable
- Reaffirm that the interviewee is happy for the interview to audio-recorded and anonymised quotes could be used in future reports or publications
- Check that the interviewee understands the reason for the interview and the role of the researcher
- Provide an opportunity for the interviewee to ask any questions
- State the interviewer may make notes during the interview

A. Background

1. Could you tell me what organisation you work for and what your current job role is?

- HMPPS – national, regional, local?

- Establishment based – what type of prison/category/population?

- Changed over the last few years/since smoke-free policy implemented?

B. Smoke-free prison policy

2. How are you/were you involved in the introduction of smoke-free policies (complete/partial) in prisons in England?

- Are you still involved – in what capacity?

3. Could you offer your reflections upon how the (complete and/or partial) policies were introduced?

- either establishment level, regionally, nationally? closed (complete) or open prisons (partial)?

- what do you think the benefits have been to the prison service going smoke-free?

- have there been any problem/unforeseen consequences/continuing issues with the smoke-free prison policy - if so, can you see these being resolved, how?

4. What impact, if any, do you think the policies have had on prisoners long term cessation and remaining smoke-free on release/transfer to open conditions?

- could you estimate relapse rates to smoking tobacco on release / transfer to cat D? As a percentage or more/less than half?

- what do you think the biggest predictors would be for those relapsing to smoking on release / transfer to cat D?

- any reasons why some prisoners do/do not return to tobacco?

C. Managing nicotine addiction in prisons

5. Could you talk me through what is available to prisoners to manage their nicotine addiction whilst in prison/within your prison for people

- is this the same for people who would like to stop smoking?

- from reception to release – is any help available for those moving to open prisons where smoking or permitted or release

6. What does the smoking cessation service in prison offer?

- when is it offered – reception and throughout a prisoners sentence?

-forms of NRT/behavioural support/e-cigarettes supported? – for how long?

- how does it currently run /could it be enhanced in any way?

- are there waiting lists for cessation? If so, how long are they?

7. Could you tell me about current practices around the use of e-cigarettes in prison?

- what e-cigarettes are offered on arrival and what can prisoners then purchase throughout their sentence?

- where in prison can prisoners use e-cigarettes – is there a national/regional/local guidance of this?

- what percentage of prisoners do you think are using them?

- how effective are they for a) abstinence whilst in prison b) quit attempts in prison.

- are prisoners allowed to take their e-cigarettes home after release or move to a cat D prison?

D. Other

8. Do you have any other questions or points that you would like to make about anything we have discussed today?

- Is there anything you’d like to raise which we have not spoken about which you think is important in relation to smoking in prison?

Thank participant for their time and answer any further questions in relation to the study?

**Supplementary File 4** . Thematic map illustrating the relationship between themes

No link
